# Metabolic gestational age assessment in low resource settings: a validation protocol

**DOI:** 10.12688/gatesopenres.13155.2

**Published:** 2021-01-28

**Authors:** A. Brianne Bota, Victoria Ward, Stephen Hawken, Lindsay A. Wilson, Monica Lamoureux, Robin Ducharme, Malia S. Q. Murphy, Kathryn M. Denize, Matthew Henderson, Samir K. Saha, Salma Akther, Nancy A. Otieno, Stephen Munga, Raphael O. Atito, Jeffrey S. A. Stringer, Humphrey Mwape, Joan T. Price, Hilda Angela Mujuru, Gwendoline Chimhini, Thulani Magwali, Louisa Mudawarima, Pranesh Chakraborty, Gary L. Darmstadt, Kumanan Wilson

**Affiliations:** 1 Clinical Epidemiology Program, Ottawa Health Research Institute, Ottawa, ON, Canada; 2 Department of Pediatrics, Stanford University School of Medicine, Stanford, CA, USA; 3 Newborn Screening Ontario, Children's Hospital of Eastern Ontario, Ottawa, ON, Canada; 4 Child Health Research Foundation, Mizapur, Bangladesh; 5 Kenya Medical Research Institute (KEMRI), Center for Global Health Research, Kisumu, Kenya; 6 Department of Obstetrics and Gynecology, UNC School of Medicine, Chapel Hill, NC, USA; 7 UNC Global Projects Zambia, Lusaka, Zambia; 8 Department of Paediatrics and Child Health, University of Zimbabwe, Avondale, Zimbabwe; 9 Department of Obstetrics and Gynaecology, University of Zimbabwe, Avondale, Zimbabwe; 10 Department of Medicine, University of Ottawa, Ottawa, Canada; 11 Bruyère Research Institute, Otttawa, Canada

**Keywords:** gestational age, newborn screening, preterm birth, machine learning, prediction modeling

## Abstract

Preterm birth is the leading global cause of neonatal morbidity and mortality. Reliable gestational age estimates are useful for quantifying population burdens of preterm birth and informing allocation of resources to address the problem. However, evaluating gestational age in low-resource settings can be challenging, particularly in places where access to ultrasound is limited. Our group has developed an algorithm using newborn screening analyte values derived from dried blood spots from newborns born in Ontario, Canada for estimating gestational age within one to two weeks. The primary objective of this study is to validate a program that derives gestational age estimates from dried blood spot samples (heel-prick or cord blood) collected from health and demographic surveillance sites and population representative health facilities in low-resource settings in Zambia, Kenya, Bangladesh and Zimbabwe. We will also pilot the use of an algorithm to identify birth percentiles based on gestational age estimates and weight to identify small for gestational age infants. Once collected from local sites, samples will be tested by the Newborn Screening Ontario laboratory at the Children’s Hospital of Eastern Ontario (CHEO) in Ottawa, Canada. Analyte values will be obtained through laboratory analysis for estimation of gestational age as well as screening for other diseases routinely conducted at Ontario’s newborn screening program. For select conditions, abnormal screening results will be reported back to the sites in real time to facilitate counseling and future clinical management. We will determine the accuracy of our existing algorithm for estimation of gestational age in these newborn samples. Results from this research hold the potential to create a feasible method to assess gestational age at birth in low- and middle-income countries where reliable estimation may be otherwise unavailable.

## Background

Reliable estimates of gestational age are critical in determining population burden of preterm birth, and informing policies for resource allocation and prioritization of interventions in low resource settings. In many low- and middle-income countries, maternal access to quality antenatal care (ANC) may be limited. In particular, gestational dating ultrasound is not routinely available and thus gestational age is often estimated using less accurate methodologies such as date of last menstrual period (LMP), symphysis-fundal height or postnatal examination of the infant’s biophysical characteristics ^1– 3^. The need for new methods to accurately assess gestational age in low resource settings has been identified as critical to ongoing efforts to improve global data on the burden of preterm birth ^4, 5^.

In addition, accurate estimates of gestational age allow for distinction between preterm infants and those who are small for gestational age (SGA). This is an important difference when allocating resources for maternal and infant healthcare and ensuring the availability of appropriate interventions. The medical needs and achievement of developmental milestones may significantly differ between preterm and SGA infants; thus, accurately identifying at-risk infants at birth is important for informing the course of their postnatal care.

One component of our research group has developed a metabolic gestational age algorithm using biomarker data obtained from routine newborn metabolic screening in a cohort of nearly 150,000 infants born in Ontario, Canada. This approach has been previously validated in cohorts in Bangladesh and Zambia. In the Bangladeshi cohort, the model correctly estimated ultrasound-validated gestational age to within one week in the majority of infants, and within two weeks in 90–95% of infants using heel prick and cord blood metabolomic markers ^6^. We have since developed and internally validated a second generation of models using a machine learning algorithm and have conducted external validation of model performance in newborn data from two cohorts from Zambia and Bangladesh ^7^. The successful use of cord blood in estimating gestational age is of particular interest as we encountered barriers to heel prick sample collection in these populations. In particular, it was difficult to collect the heel prick sample in the recommended 24–72 hour window due to early hospital discharge. We also encountered parental reluctance to subject their infants to a heel prick sample collection, especially in preterm infants ^6^.

While our previous work, and that of others ^5, 7^, suggests potential value in using dried blood-spot-derived analytes for gestational age assessment, some key questions need to be answered before attempting to utilize this approach widely as a surveillance tool for preterm birth:

1. Is the approach feasible for implementation at primary birthing facilities in low-resource settings?
2. Does application of the approach at primary birthing facilities provide an accurate estimate of the preterm birth rate in the population?
3. What is the accuracy of metabolic gestational age assessment using cord blood relative to infant heel-prick blood?
4. Can the approach distinguish SGA from appropriate-for-gestational age (AGA) and large-for-gestational age (LGA) infants?
5. Can the performance of the algorithm be enhanced by including additional measures, such as anterior lens vascularity assessment, Ballard scoring, and anthropometric measurements?

## Methods

### Objectives

The goal of this study is to pilot a program that derives gestational age estimates from dried blood spot samples (heel-prick or cord blood) collected from a population-representative cohort in health and demographic surveillance sites in low-resource settings.

If successful, this will achieve the following objectives:

Primary objectives

1. Assess the feasibility of large-scale implementation of metabolic gestational age screening.
2. Estimate population-level burdens of preterm birth within select low-resource areas.

Secondary objectives

1. Estimate population-level burdens of SGA infants within low-resource settings through validation of a newly developed algorithm that simultaneously classifies infants as SGA/AGA/LGA while estimating gestational age.
2. Estimate population-level burdens of treatable, rare metabolic diseases.
3. Identify and treat infants at risk for these treatable life-threatening/altering metabolic diseases
4. Generate a large international newborn metabolic dataset that could be leveraged for future preterm birth research

At a population level, utilization of metabolic analysis for gestational age dating could support surveillance of the burden of preterm birth and research into the development of effective resource management strategies. We will seek to determine whether this is feasible in low-and middle- income settings.

### Ethics approval

This study was approved by the Ottawa Health Sciences Network Research Ethics Board (20180330-01H), Children’s Hospital Of Eastern Ontario Research Ethics Board (18/58X), the Stanford University School of Medicine Institutional Review Board (44656), the Kenya Medical Research Institute Scientific and Ethics Review Unit (SSC 2880), the University of Zambia Biomedical Research Ethics Committee (015-06-17), the Bangladesh Institute of Child Health (BICH-ERC-01-01-2019), and the Research Council of Zimbabwe (03744).

### Project funding

The Ottawa Hospital Research Institute (OHRI) has received funding from the Bill & Melinda Gates Foundation to cover project coordination costs in Ontario, Canada, including but not limited to sample analysis, gestational age estimation model execution and project management (OPP1184574). Stanford University received funding from the Bill & Melinda Gates Foundation to operationalize the project including site selection, protocol training, subcontracting to international sites, oversight and communication with international sites (OPP1182996).

### Study setting

This project is led by the OHRI with services provided by Newborn Screening Ontario (NSO), in partnership with the Department of Pediatrics, Stanford University and supported by the Bill & Melinda Gates Foundation. Sample collection sites include: Mirzapur, Bangladesh led by the Child Health Research Foundation; Kisumu, Kenya led by a team from the Kenya Medical Research Institute (KEMRI) and Center for Disease Control and Prevention (US CDC) collaboration; Lusaka, Zambia led by UNC-GPZ; and Harare, Zimbabwe led by researchers from University of Zimbabwe.

### Site selection

Investigators at Stanford University were responsible for identifying and training international partners in sample collection at facilities located in South Asia and sub-Saharan Africa. Identification of eligible site partners was based on capacity for sample collection from population representative cohorts and the ability to collect samples from both hospital and primary birthing facilities in order to evaluate whether metabolic gestational age dating approaches were valid estimates of population-level preterm birth rates.

We evaluated 19 sites for inclusion in the study. Sites were deemed acceptable if they met the following specific criteria: early pregnancy registration, ultrasound capacity, ability to obtain newborn heel-prick and cord blood samples, capacity to store and ship blood spot cards and cord blood samples, ability for patient follow up and demographic surveillance, and ability for follow-up testing for screen positive results for treatable metabolic diseases. Four sites were identified that broadly met our inclusion criteria. All sites were trained on cord blood and heel-prick derived dried blood spot collection. 

### Sites

***Mizapur, Bangladesh.*** The Child Health Research Foundation is a demographic surveillance site (DSS) with births occurring at the Kumudini Hospital, local clinics, and at home. The DSS sites covers 374 km ^2^, with a population of close to 300,000 people. One data collector covers approximately 400 households and visits every four months to collect demographic information. The preterm birth rate at this site is estimated to be 18–19% based on LMP. Half of the study population will be enrolled at the time of birth at the birthing center at the Kumudini Hospital, and half will be enrolled at 20 or before weeks’ gestation through the DSS to allow for early trimester ultrasound validation. Women enrolled at 20 weeks gestation will be transported to Kumudini Hospital for ultrasound examination. Heel prick samples will be collected for all infants, and cord blood samples will also be collected for infants born at Kumudini Hospital. An estimated 840 cord and 1455 heel prick samples will be collected from this site over an estimated 12-month period.

***Kisumu, Kenya.*** The Kenya research center is located in Kisumu, at the KEMRI Center for Global Health Research. Field research sites are located in Siaya county where a maternal-infant surveillance platform hosting a prospective cohort of pregnant women and their infants is implemented from two community hospitals; Siaya County Referral Hospital (SCRH) and Bondo sub-County Hospital (BSCH). The SCRH is the main referral hospital for the county region. Bondo Hospital, located 23 km south, serves as a primary birthing center and enrollment site for the community. Prior studies have estimated the preterm birth rate to be 19% by a combination of LMP and ultrasound. Participants are enrolled at their first ANC visit or by research nurses or through trained community health workers (CHWs) who administer home-based urine pregnancy testing and refer pregnant women to the study clinics for further screening and enrollment. Eligible participants at this site include pregnant women 15–49 years of age, coming from a 10 km radius of the research facility, willing to deliver in the research hospital and not planning to relocate/migrate within one year of enrollment into the surveillance program. Women are typically enrolled prior to 20 weeks’ gestation at which time they undergo ultrasound, and are offered treatment for common illnesses including malaria, urinary tract infections and sexually transmitted infections. It is expected that 10% of babies will be born at home, with infants evaluated within 72 hours. Expected enrollment is approximately 780 cord and heel samples with paired ultrasounds for all women collected over one year.

***Lusaka, Zambia.*** Building on prior work of our pilot project nested in the ZAPPS-1 study, we partnered with UNC-GPZ to implement metabolic analysis for the participants of two existing study cohorts: the Zambian Preterm Birth Prevention Study (ZAPPS-2) and Improving Pregnancy Outcomes with Progesterone (IPOP) ^8, 9^. This site has an estimated preterm birth rate of 15% by ultrasound ^10^. Inclusion criteria for ZAPPS-2 and IPOP are women 18 years of age or older, residing in Lusaka with no plans to relocate. Women in the ZAPPS-2 cohort are HIV uninfected, whereas women enrolled in the IPOP study are HIV-1 positive and receiving antiretroviral therapy. HIV status and anti-retroviral therapy will be accounted for in the analysis phase. All enrollees are recruited prior to 24 weeks’ gestation in this cohort, and ultrasound occurs at the time of enrollment. Enrollment occurs over eight district clinics with delivery in the Lusaka University Hospital, creating a population representative sample for peri-urban Zambian women. The goal sample size for this site is estimated to be 600 cord and heel samples, all paired with ultrasound. Sample collection is expected to occur over one year.

***Harare, Zimbabwe.*** The Zimbabwe site consists of two primary birthing clinics and the referral hospital with a large volume of community and hospital deliveries. Most pregnancies are registered after 24 weeks of gestation. Ultrasound is not routinely available unless complications are identified or by self-pay in a private clinic. Extensive laboratory capacity exists for follow-up testing of screen positive results; however, mass spectrometry is not available. The estimated preterm birth rate is 12% based on LMP. Half of participants in the Zimbabwe cohort will be enrolled at birth from the referral hospital and half will be enrolled during their first ANC visit at primary care clinics, at which time they will undergo ultrasound, yielding a total sample size of. Participants enrolled at primary care clinics will be transferred to the referral hospital if they go into preterm labour. The target sample size for this site is approximately 1000 participants (1000 heel and 500 cord samples) and we expect sample collection to occur over nine months.

### Sample size

Determining sample size requirements for an each site was guided by Steyerburg (2010) ^11^ and based on typical birth rates at each site and number of ultrasounds that could be obtained. A pragmatic sample size of n=20 was planned for the smallest subgroup in which model performance metrics were to be calculated (ie.GA <34 weeks). This ensured sufficient sample size to calculate reasonably robust summary (i.e. mean and CI) of the reported performance metrics in each planned subgroup. 

### Patient consent

At all sites, parental informed consent will be sought prior to enrollment in the study, and consent forms will be available in local languages. All liveborn infants will be eligible for inclusion in this study. Women will be enrolled by research nurses during the first ANC visit or at delivery (depending on site enrollment criteria). Participants will be made aware that participation is voluntary, and the risks and benefits of enrollment will be discussed. Those choosing not to participate will continue to receive ANC and treatment according to local clinical standards. Participants will be assured that all reasonable measures will be taken to protect their personal information and that of their newborn. Participants enrolled at the Bangladesh, Zambia, and Kenya sites will also consent to storage and future use of their samples for perinatal research under authorization of presiding institutional committees.

Consenting parents will be informed that heel-prick collection may cause temporary discomfort to their newborn and will also be informed of the possibility that their child may be identified to be at risk of one or more treatable metabolic conditions. Participants will be informed that such incidental findings will be communicated to the study investigators and recommendations will be made to confirm diagnoses and help guide any necessary treatment of their infant. At the time of consent, participants will be informed of their right to request the full details of the screening results for their newborns.

### Collection of newborn screening specimens

**Figure 1.**
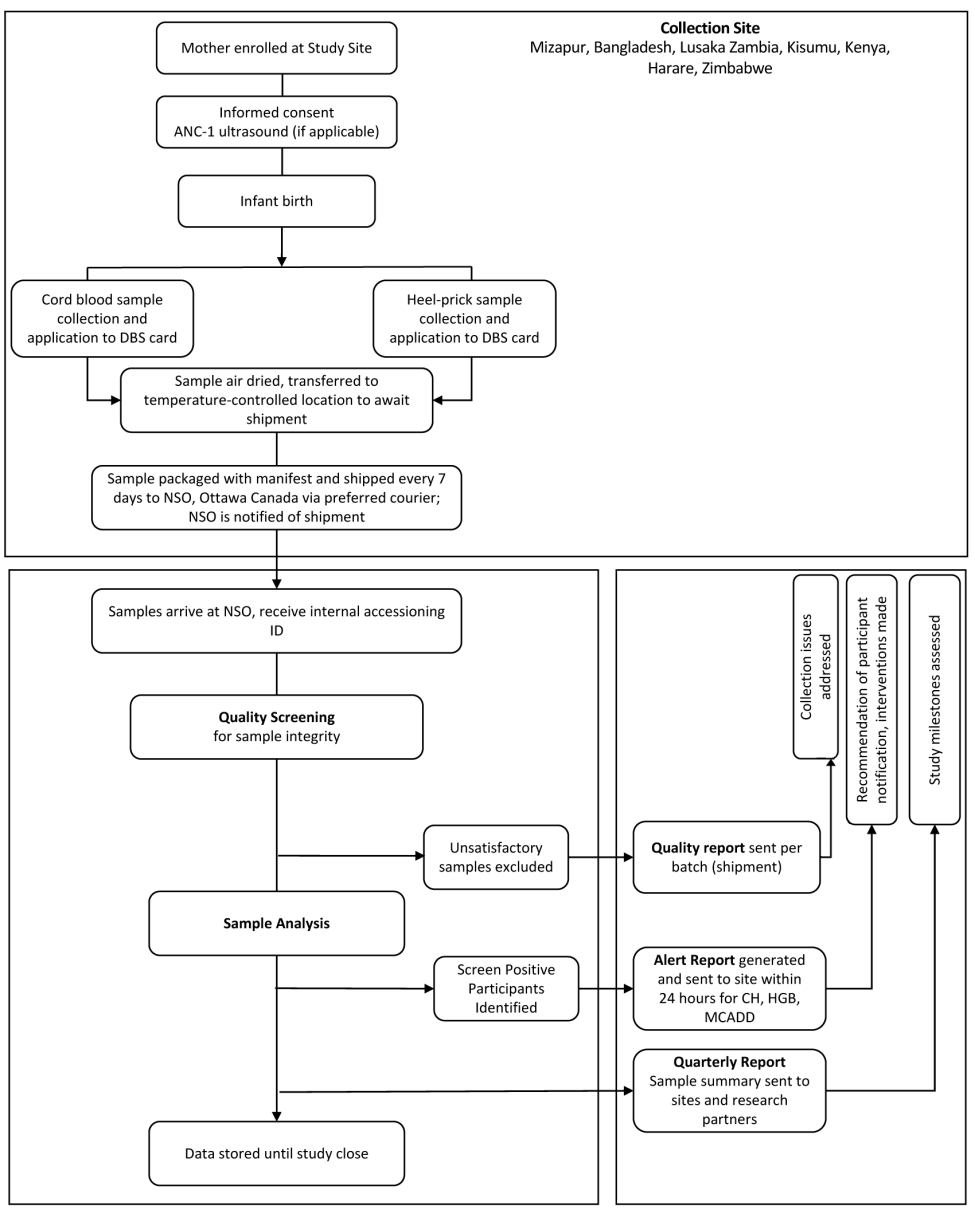
Study Workflow. Participants are enrolled in the research study, and samples collected at the collection sites are sent weekly via preferred courier to Newborn Screening Ontario (NSO), Ontario, Canada for analysis. Reporting procedures from the NSO to the collection site include provision of reports on sample quality, on newborns’ risk of congenital hypothyroidism (CH), medium-chain acyl-CoA dehydrogenase deficiency (MCADD) and haemoglobinopathies (HGBs) and quarterly reports summarizing study progress. Adapted from 12 under a CC BY 4.0 license. ANC, antenatal care; DBS, dried blood spot; NSO, Newborn Screening Ontario; CH, congenital hypothyroidism; HGB, hemoglobinopathies; MCADD, medium-chain acyl-CoA dehydrogenase deficiency.

Study personnel will collect, handle and transport heel-prick and cord dried blood spot samples from participants according to study-specific standard operating procedures ( Figure 1). Sample collection procedures have been published previously ^12^. In brief, cord blood will be collected within 30 minutes of delivery of the placenta into an uncoated, sterile syringe. Blood from the syringe (4–5 drops) will be applied to designated filter paper within pre-printed circles. Newborn heel-prick samples will be collected ideally between 24–72 hours after birth or prior to discharge if the newborn is released from hospital within 24 hours of delivery. The newborn’s heel will be warmed at the identified skin-puncture site to promote increased blood flow. The puncture site will be cleaned and air-dried. A sterile lancet or heel incision device will be used to puncture the lateral aspect of the plantar surface of the newborn’s heel. The first small drop of blood formed will be cleaned away and the formation of a second large drop of blood will be encouraged by intermittently applying gentle pressure to the newborn’s lower leg and heel. A filter card will be applied gently against the blood drop to soak through. Each preprinted circle on the filter card will be filled by subsequently formed drops of blood. Following heel-prick sample collection, the newborn’s foot will be elevated above the body and a sterile gauze pad or cotton swab will be pressed against the puncture site until the bleeding stops. No more than 500µL of blood of each sample type (heel prick or cord) will be required for this study. To reduce newborn discomfort during the heel prick sample collection, mothers will be encouraged to hold the baby close or breastfeed the baby before and during the procedure.

Labels with unique site-specific de-identified participant identifiers (ID) will be affixed to each filter card at the time of collection. Samples will be air-dried for 3–4 hours or overnight after which they will be transported to a designated secure study location (hospital office or laboratory) and stored in a room temperature setting prior to shipping. Samples will be shipped at room temperature from the collection sites to NSO every seven days by a preferred courier service. We have previously found samples to be stable for up to two months at room temperature in climate-controlled conditions ^13^. Appropriate shipping standards will be communicated to the collection sites to minimize the risk of compromised sample integrity during shipping.

An electronic shipment manifest containing clinical and demographic information essential for clinical interpretation of newborn metabolic profiles will be relayed to NSO and OHRI ahead of shipping and included as a hard copy in the shipment ( Table 1). Upon receipt of the samples at NSO, the sample manifest will be cross-referenced against the physical sample cards. Any discrepancies will be resolved through follow-up with the collection site. Secondary accession numbers will be applied for use by internal NSO systems.

**Table 1.** Data variables required to support newborn screening analysis.

Variable	Values
De-identified participant ID	
Sample type	Cord or heel
Application method to filter card	Direct, tube, syringe
Sex	Male, female, ambiguous
Birthweight	grams
Multiple birth	Yes/no; if yes, baby 1,2,3 or a,b,c
Feeding status	Breast, TPN, formula, NPO
Packed red blood cell transfusion	Yes/no: if yes, date of latest transfusion
Gestational age	If available; weeks + days
Date and time of birth	
Date and time of sample collection	

TPN, total parenteral nutrition; NPO, nil per os (nothing by mouth).

### Newborn screening analysis

NSO is Ontario’s provincial newborn screening program, located at the Children’s Hospital of Eastern Ontario Research Institute (CHEO RI). All samples collected will be directed to NSO for laboratory analysis. NSO is equipped with sufficient personnel and laboratory resources to accommodate the inclusion of international clinical and research samples into the organization's standard workflow. The organization is currently providing newborn screening services to remote communities in other Canadian provinces/territories, and has piloted international screening services ^14^. 

### Quality management of samples

NSO will refer to organization and international standards to guide quality management of samples ^15, 16^. Each sample received will be reviewed for specimen quality and quantity. Newborn screening test calculations assume that the blood is evenly distributed within the circle and completely saturates both sides of the filter paper, and so a satisfactory newborn screening specimen will have blood fully soaked through to the back of the filter paper. Only satisfactory samples will be used for study analysis. Samples can be deemed unsatisfactory for several reasons as outlined in Table 2. If the sample quality is unsatisfactory due to insufficient quantity of blood, the sample will be excluded from analysis or undergo partial analysis.

**Table 2.** Sample quality screening criteria

Parameter of sample quality	Quality	Action for analysis
Acceptable	Satisfactory	Include
Blood spot collection paper expired	Unsatisfactory	Exclude
Blood spots appear clotted or layered	Unsatisfactory	Exclude
Blood spots appear diluted	Unsatisfactory	Exclude
Blood spots appear scratched or abraded	Unsatisfactory	Exclude
Blood spots appear damaged	Unsatisfactory	Exclude
Blood spots are supersaturated	Unsatisfactory	Exclude
Blood spots are wet/discoloured	Unsatisfactory	Exclude
Blood spots exhibit serum rings	Unsatisfactory	Exclude
Quantity of blood insufficient	Unsatisfactory	Review decision *

* *If the sample quality is unsatisfactory due to the insufficient quantity of blood, the sample will be reviewed at time of receipt and will be excluded from analysis or undergo partial analysis.*

NSO will generate Quality Reports summarizing the quality of samples received in each shipment. These reports will be sent electronically to the collection site and used to address issues of sample collection, handling and storage prior to shipment. The Report will summarize the Batch ID for the batch of samples received, specimen details and the quality of the samples, as outlined in Table 2.

### Sample analysis

Standard practice at NSO is to screen each sample for metabolites indicative of risk for approximately 30 treatable conditions. Full-panel analyses as per standard NSO practice will be executed on samples when sample quality is satisfactory.

### Quarterly reporting

Quarterly Reports summarizing study progress will be generated by NSO and electronically shared with the study sites, Stanford University and OHRI. Quarterly Reports will include a summary of the analytical results collected up to the end of that quarter, and summarize the total number of samples received, sample collection data (time of collection), newborn characteristic data (gestational age, sex, multiple birth etc.) and the number and distribution of samples excluded from analysis based on ‘unsatisfactory’ quality.

### Management of incidental findings

The conditions screened as part of the NSO screening panel are rare. It is possible that an infant will be identified to be at high risk for one of these screened conditions through sample analysis. Such risk poses clinical and ethical considerations for gathering newborn screening data for research purposes. While this study involves newborn screening, this study is not being undertaken as a newborn screening initiative and is designed explicitly as a non-interventional study. 

Real time notification of incidental findings will be made for conditions that can be feasibly treated at the collection sites. Sample integrity, potential for confirmatory testing and potential for intervention were all taken into consideration when determining the feasible management of incidental findings, and our approach and rationale have been described in detail elsewhere ^6, 12, 17^. Based on the above considerations, three high-priority conditions have been identified for real-time reporting: congenital hypothyroidism (CH), hemoglobinopathies, and medium-chain acyl-CoA dehydrogenase deficiency (MCADD).

In brief, in the event that an infant screens positive for a priority condition, second-tier analysis of samples, including repeat confirmatory testing, CFTR mutation analyses, TBX1 and purine profile assays, will be executed as per a modified workflow based on standard NSO practice, provided sufficient sample is available from the original blood spot card. Electronically encrypted Alert Reports will be provided as soon as analysis is complete, and the screening results have been interpreted. The Alert Report will indicate the disease for which the infant is at high risk, the flagged analyte, the analyte level, and the reference ranges and thresholds for the flagged analyte. To ensure that communication of clinically relevant findings between investigators is successful, the collection site will confirm receipt of all Alert Reports within 24 hours. The report will recommend that positive screen results be communicated to the participant’s family, and that appropriate follow-up testing be conducted along with any necessary medical intervention, implemented in accordance with local treatment guidelines. The collection site will report back to the lead site with a completed Alert Report identifying confirmatory testing and medical follow-up.

### Data management

Upon completion of sample collection, sites will provide OHRI and Stanford with a detailed data collection form with maternal and neonatal covariates required for model validation and analysis ( Table 3). The data will be paired with matching newborn screening data provided by NSO into a single, combined dataset to be used for analysis. Once the primary analytic goals of the project have been met, these data will be stripped of patient identifiers and made available as a shared dataset by site and shared with the appropriate participating institutions in the pursuit of open collaborative research. A common data use agreement will be generated to govern the sharing and use of the data.

**Table 3.** Maternal and neonatal covariates to be included for the study.

Neonatal characteristics	Date of ANC-1 ultrasound Gestational age at time of ultrasound (weeks + days) Birth weight (grams) Gestational age (weeks + days) Date and time of birth Date and time of sample collection Feeding status (breast, TPN, formula, NPO) Transfusion (yes, no; if yes, date of latest transfusion) Multiple births (yes, no; if yes, baby a, b, c or 1, 2, 3) Low birth weight, intrauterine growth restricted, small- or large-for-gestational age Mode of delivery (Caesarian section, vaginal: spontaneous, practitioner induced) Presentation at time of delivery Apgar scores ALCV image capture Ballard score
Maternal characteristics	Age (years) Body mass index (weight (kg)/ (height (m))2) Parity and gravidity Smoking status Alcohol consumption (if available) Diabetes Hypertension HIV status (if known) Viral load (if applicable)

ANC, antenatal care; TPN, total parenteral nutrition; NPO, nil per os (nothing by mouth); ALCV, anterior lens capsule vascularity.

The research team at the Ottawa Hospital Research Institute (OHRI) will lead primary data analyses for the generation of gestational age, preterm birth and SGA estimates, and all regulatory activities required at OHRI and CHEO RI, including but not limited to research contracts, data/material sharing agreements and ethics approvals. Each site will be provided with copies of their own cohort data for use in future analyses in consultation with the overall group.

### Statistical analysis

***Model-based gestational age estimation and classification of preterm birth, SGA/AGA/LGA using clinical covariates and newborn screening metabolites.*** Newborn gestational age will be estimated from models derived using multivariable regression coupled with elastic net regularization and including the following covariates:

1. Model 1: Birth weight, sex, multiple birth status and pairwise interactions
2. Model 2: Sex, multiple birth status, newborn screening analytes and pairwise interactions
3. Model 3: Birth weight, sex, multiple birth status and newborn screening analytes

Classification of preterm birth, as well as SGA/AGA/LGA is conducted using an iterative multitask classification machine learning algorithm that simultaneously derives classification models for both preterm birth status and SGA/AGA/LGA and uses the prediction of one to inform the prediction of the other. These models include birthweight, sex, multiple birth status and newborn screening analytes as predictors, similar to Model 3 above. The model classifying preterm birth status will also use the prediction of SGA/AGA/LGA from the other model, and vice versa, improving the accuracy of both predictions, and will also employ an elastic net or lasso regularization approach similar to gestational age estimation models. Gestational age size classification (SGA/AGA/LGA) will be based on the INTERGROWTH-21 standards ^18^.

Models were trained and internally validated in independent training and validation/test cohorts of infants from Ontario, Canada. These pretrained models can then be applied to the data for infants from the external cohorts to produce gestational age estimates. In order to evaluate the accuracy of gestational age estimates calculated from the models, the model estimate will be compared to the ultrasound reference gestational age for each infant, and the residual error calculated (model gestational age minus reference gestational age). These residuals can be squared, absolute value taken, summed and averaged to calculate different metrics including: mean square error (MSE); standard error of estimation, also known as root mean square error (RMSE); and mean absolute error (MAE), which is the average of the absolute value of the residual across all subjects (or subsets of subjects). Additionally, we will calculate the proportion of model-derived estimates that fall within ± 1 and ± 2 weeks of reference ultrasound gestational age for those samples with gestational age available. MAE will be the main performance metric we will use to evaluate model accuracy, but multiple metrics will be calculated and reported to facilitate comparisons to other models developed by our group and others. Model-derived frequency of preterm birth will be compared to the preterm birth frequency based on reference ultrasound gestational age by dichotomizing the model and reference gestational age values, where preterm is defined as gestational age <37 weeks and term is defined as gestational age ≥37 weeks.

For classification models predicting preterm birth status and SGA/AGA/LGA, we will report area under the ROC curve, as well as sensitivity/specificity and positive predictive values. We will assess model calibration by constructing and visually inspecting calibration plots of and through calculation of the calibration slope and intercept for a regression of model gestational age vs. reference gestational age, and for predicted vs actual probabilities of preterm birth and SGA/AGA/LGA for classification models.

***Assessment of other gestational age measures.*** We will collect additional assessments of gestational age, including anterior lens vascularity, Ballard score, anthropometric measures, LMP and first trimester ultrasound to compare the accuracy of gestational age estimates with our algorithm.

### Dissemination

Knowledge Translation will be facilitated through the relationships between researchers, funding organization, and the communities involved in the project. Our findings will be disseminated through publication in peer-reviewed Open Access journals and presentations at academic conferences. We will promote awareness of our findings through press release within our institutions, locally and abroad. The data is collectively owned by OHRI, CHEO and Stanford. Third party de-identified data sharing is possible with the expressed written agreement of all applicable parties and with the development of the appropriate Data Sharing Agreements.

### Study status

All ethics approvals and contracts are in place between all partners. NSO began receiving samples from Zambia, Bangladesh and Kenya in April 2019 and sample collection is nearing completion at these sites. For Kenya and Bangladesh we have not observed the same hesitancy to perform heel prick blood sampling as in our previous studies ^6^. Sample collection in Zimbabwe was planned to start in March 2020 but study initiation was delayed due to the COVID-19 global pandemic.

## Impact of the project

Preterm birth continues to be a leading cause of mortality in children under five ^19^ and being born too early or too small has long-term health and economic consequences ^20^. Accurate estimates of the population burden of preterm birth are critical to inform resource allocation and prioritize interventions for preterm birth. This project will provide critical insight into the feasibility of the large-scale implementation of metabolic gestational age screening in low resource settings and will inform the logistical requirements and feasibility of scaling up our protocol and algorithms for population surveillance of preterm birth and SGA estimates in low-resource settings.

By comparing estimates obtained from primary birthing centers and DSS, insights will be gained into the feasibility of obtaining accurate estimates of population-level preterm birth rates through enrollment at primary birthing centers. We will also compare the accuracy of estimates of gestational age derived from cord blood samples and infant heel-prick samples, which will be important for accommodating local preferences for specimen collection method in future studies.

This project will also generate a large international database of newborn screening data that could be leveraged for future preterm birth research.

Our protocol for screening of certain diseases will both inform on the prevalence of these conditions and identify and treat afflicted infants for potentially life-threatening/life altering conditions that would have otherwise gone undiagnosed. In our previous prospective cohort study, 61 infants were identified with congenital hypothyroidism and hemoglobinopathies in a cohort of 1661 samples from a rural community in Bangladesh ^18^. In the current cohorts, diagnostic capabilities and treatment are available at all sites. Screening cut-offs are set higher for international sites compared to normal NSO diagnostic criteria to allow for blood spot screening to have a high positive predictive value.

At present, options for the treatment of either SGA or preterm infants remain limited in low- and middle-income countries. The inability to distinguish preterm and SGA infants due to unreliable gestational age estimation precludes different treatment strategies for these two populations. Interventions, such as Kangaroo-Mother Care, are targeted at low birth weight infants and thus likely capture both SGA and pre-term infants. Better distinction between these two populations in low-resource settings could contribute to more targeted approaches and potentially better outcomes.

We have conducted a cost-effectiveness analysis of our previous work in Bangladesh ^6^ which further informs the feasibility of this approach for preterm birth classification. Coyle *et al*. found that the incremental annual cost of adopting the metabolic algorithm was $100,031 ($120, 496 including start-up costs). This amounts to $11,542 per preterm infant correctly identified or $688 per SGA infant correctly identified. The cost of the algorithm is potentially improved when a targeted approach is used by screening infants between 2500-3600g, as infants <2500g can be correctly identified using a basic algorithm and infants >3600g are never preterm ^21^. Ultimately, decision makers and potential funders will have to consider the cost of various interventions compared to their benefits when deciding whether to implementing metabolic screening for gestational age.

Finally, this study will also inform the proper sample handling, storage and processing procedures for accurate assessment of preterm birth and newborn screening and thus could inform the feasibility for implementing newborn screening practices in low-resource settings either on site or with international partners.

In summary, this protocol provides details on an important prospective cohort study to estimate gestational age in low resource settings and will provide valuable insight into the utility of newborn screening analytes in evaluating preterm birth rates in low-resource settings.

## Data availability

No data are associated with this article.
