# Supplementary material for: Metabolic gestational age assessment in low resource settings: a validation protocol
Source: Gates Open Res. 2021 Jan 28;4:150. Originally published 2020 Oct 2. [Version 2] doi: 10.12688/gatesopenres.13155.2 (PMC7801859; doi:10.12688/gatesopenres.13155.2)
Supplement: Peer review history

# Peer review: Reviewer response for version 1

**DOI:** 10.21956/gatesopenres.14350.r30180

**Published:** 2021-01-22

**Authors:** Zilma Silveira Nogueira Reis

**Affiliations:** 1 Center of Health Informatics, Universidade Federal de Minas Gerais, Belo Horizonte, Brazil

This paper presents a study protocol to validate the metabolic gestational age assessment using cord blood relative to infant heel-prick blood in low-resource settings. Current challenges for gestational age assessment were described, as well as prior steps of this new approach. This feasibility study started already, expecting a large-scale implementation of metabolic gestational age screening. If it has not started, please inform the expected date.

 Low-resources birth settings claim for prompt and easy-to-implement solutions with the potential to indicate neonatal risk, facing unknown or unreliable gestational age. I consider this program a possible response to such complaints.

 My questions and suggestions:

1. Secondary objective number 3 is unclear to me - “Identify and treat infants at risk for these treatable life-threatening/altering metabolic diseases.” During the first 48 hours of life, very early blood samples are associated with false-negative screening for diseases as congenital hypothyroidism, phenylketonuria. Newborn heel-prick samples will be collected ideally before discharge, mostly within 24 hours of delivery. How will researchers deal with this issue?
2. How will the study conduct treatment of rare neonatal diseases screened in low-income countries?
  • Is the study design appropriate for the research question?
   
  - Yes, I consider the study design appropriate for the primary objective. I suggest the study registration in the WHO Clinical Trials Platform.
    • Are sufficient details of the methods provided to allow replication by others?
3. In methods, details of antenatal ultrasound assessment are not clear. Ultrasound dating uses fetal size to determine gestational age based on the assumption that fetuses grow at the same rate in early pregnancy. Which fetal growth curve will be used, and who will assess fetuses with the ultrasound?
4. Regarding SGA, AGA, and LGA assessment using algorithms, what is the reference curve for fetal growth standardized in the study?
5. Ballard's score inter-observer error cannot be overlooked, and the exam demands health professional experience. How will the study reduce the bias of the observer's expertise?
6. About the sample size, is there any estimate to achieve significant results?

Is the study design appropriate for the research question?

Yes

Is the rationale for, and objectives of, the study clearly described?

Yes

Are sufficient details of the methods provided to allow replication by others?

Partly

Are the datasets clearly presented in a useable and accessible format?

Not applicable

Reviewer Expertise:

I am experienced in Maternal and Child Health, and Medical Informatics, acting on the following topics: medical informatics, scientific methodology, and women's health.

I confirm that I have read this submission and believe that I have an appropriate level of expertise to confirm that it is of an acceptable scientific standard.

# Peer review: Reviewer response for version 1

**DOI:** 10.21956/gatesopenres.14350.r30047

**Published:** 2021-01-11

**Authors:** Thokozile Rosemary Malaba

**Affiliations:** 1 Division of Epidemiology & Biostatistics, School of Public Health & Family Medicine, University of Cape Town, Cape Town, South Africa

This manuscript describes the pilot testing of metabolic gestational age assessment using DBS in low-income settings. GA assessment is challenging in resource-limited settings where ultrasonography is typically unavailable and routinely based on clinical assessment; as such this manuscript addresses an important public health concern by presenting a potential option for GA assessment in low-income settings.

 This is a well-written manuscript and I only have the following minor comments/queries:

**Background:**

- Repetition of the following paragraph: “while our previous work, and that of others, suggests potential value…widely as a surveillance took for preterm birth".

**Methods:**

- Perhaps the authors could provide the target sample sizes for samples from each site - the justification/calculations for each site are not clear.
- Could there be standardization of language/structure of the site descriptions in terms of the site (which facilities), site’s PTB rate, timing of enrolment, eligibility/inclusion criteria, sample size of samples vs participants?
- Site-specific comments:
  - Bangladesh
    - *“...Kumudini Hospital, and half will be enrolled at 20 weeks gestation.....to allow for first trimester ultrasound validation*”
      - Were these women enrolled **at 20 weeks **or at **20 weeks or earlier** - if the former perhaps first trimester ultrasound should be changed to early ultrasound.
  - Kenya
    - *“Participants enrolled at their first ANC visit....”*
      - *Were women enrolled at this visit enrolled regardless of gestational age? *
        - *Linked to point above “Women are typically enrolled prior to 20 weeks gestation” - what is the procedure for late presenters? Are they enrolled? If yes do they have an ultrasound assessment?*
  - Zimbabwe
    - Similar to the comment above are there any GA restrictions for women enrolled at first ANC visit?
    - “Yielding a total sample size of approximately 1000 participants”.
      - Does this mean the goal sample size is estimated to be 1000 heel and cord samples?
- *“Classification of preterm birth, as well as SGA/AGA/LGA….”*
  - Which standards will be used for classification of size for gestation age - is it an international standard like INTERGROWTH-21st or regional standards?

Is the study design appropriate for the research question?

Yes

Is the rationale for, and objectives of, the study clearly described?

Yes

Are sufficient details of the methods provided to allow replication by others?

Yes

Are the datasets clearly presented in a useable and accessible format?

Yes

Reviewer Expertise:

perinatal epidemiology, maternal and child health

I confirm that I have read this submission and believe that I have an appropriate level of expertise to confirm that it is of an acceptable scientific standard.

# Response

**Published:** 2021-01-19

**Authors:** Kumanan Wilson

**Affiliations:** Ottawa Hospital Research Institute, Canada

This is a well-written manuscript and I only have the following minor comments/queries:

**Background:**

- Repetition of the following paragraph: “while our previous work, and that of others, suggests potential value…widely as a surveillance took for preterm birth".

***Response:***

 Thank you for noticing the paragraph repetition, it appears to be an error during publication.

**Methods:**

- Perhaps the authors could provide the target sample sizes for samples from each site - the justification/calculations for each site are not clear.

***Response:***

*We have added a subsection to the methods providing further details on the sample size calculations. The estimated sample size provided for each site is the target sample size for each population. For sample size requirements for an external validation, we followed the general philosophy and guidance of Steyerburg’s Clinical Prediction Models. The estimated preterm birth rates at each site and number of ultrasounds that could reasonably be obtained were also considered. We were guided by a pragmatic target sample size of 20 observations in the smallest subgroup as this sample size is sufficient to calculate a robust summary (e.g. mean and CI) of the reported performance metrics in each subgroup of interest, for continuous GA, without requiring an unachievably large sample size. For example, if the rate of <34 weeks was 5%, this would require an overall sample size of n=400 (20/0.05) for that site.*

**

*This subsection is presented following the site selection section in the Methods.*

- Could there be standardization of language/structure of the site descriptions in terms of the site (which facilities), site’s PTB rate, timing of enrolment, eligibility/inclusion criteria, sample size of samples vs participants?

***Response:***

 W *e have structured the information, so it follows a similar pattern for each site – site details, estimated PTB, enrollment details, target sample size.*


*  *

- Site-specific comments:
  - Bangladesh
    - *“...Kumudini Hospital, and half will be enrolled at 20 weeks gestation.....to allow for first trimester ultrasound validation*”
      - Were these women enrolled **at 20 weeks **or at **20 weeks or earlier** - if the former perhaps first trimester ultrasound should be changed to early ultrasound.

***Response:***

*Thank you, we have edited this to say ‘enrolled at or before 20 weeks to allow for early ultrasound validation.*

-
  - Kenya
    - *“Participants enrolled at their first ANC visit....”*
      - *Were women enrolled at this visit enrolled regardless of gestational age?*
        - *Linked to point above “Women are typically enrolled prior to 20 weeks gestation” - what is the procedure for late presenters? Are they enrolled? If yes do they have an ultrasound assessment?*

***Response:***

*At this site, participants are typically enrolled prior to 20 weeks. Some participants are enrolled outside of the recommended range. All participants enrolled undergo ultrasound, and the date of ultrasound will be considered during analysis.*

-
  - Zimbabwe
    - Similar to the comment above are there any GA restrictions for women enrolled at first ANC visit?

***Response:***

*Ultrasound enrollment target is <20 weeks, however enrollment beyond this gestational age may occur if this is necessary to achieve sample size goals. This would be accounted for in the analysis.*

-
  -
    - “Yielding a total sample size of approximately 1000 participants”.
      - Does this mean the goal sample size is estimated to be 1000 heel and cord samples?

***Response:***

*We have updated this to add ‘…with 1000 heel and 500 cord samples*


*  *

- *“Classification of preterm birth, as well as SGA/AGA/LGA….”*
  - Which standards will be used for classification of size for gestation age - is it an international standard like INTERGROWTH-21st or regional standards?

***Response:***

*We will use intergrowth-21 standards for gestational age classification, we have added this to the statistical analysis section.*

# Peer review: Reviewer response for version 1

**DOI:** 10.21956/gatesopenres.14350.r29735

**Published:** 2020-11-02

**Authors:** Tina M. Slusher

**Affiliations:** 1 Department of Pediatrics, University of Minnesota, Minneapolis, MN, USA; 2 Pediatric Critical Care Faculty, Hennepin Healthcare, Minneapolis, MN, USA

I think this article is scientifically sound, well written and easy to follow. The only comments I have are not related to the soundness of the proposal but rather their comments that the metabolic diseases they are choosing to screen for are exceedingly rare - hemoglobinopathies like sickle cell disease are *not* exceedingly rare and my concerns about how cost effective this would be to sustain after the project in LMICs where it would be most needed. Additionally I would have considered adding G6PD testing to their batteries of tests because although not treatable per say, knowing the infants status is important over their lifetime in terms of medicines they avoid and problems they watch for.

Is the study design appropriate for the research question?

Yes

Is the rationale for, and objectives of, the study clearly described?

Yes

Are sufficient details of the methods provided to allow replication by others?

Yes

Are the datasets clearly presented in a useable and accessible format?

Not applicable

Reviewer Expertise:

NA

I confirm that I have read this submission and believe that I have an appropriate level of expertise to confirm that it is of an acceptable scientific standard.

# Response

**Published:** 2020-11-10

**Authors:** Kumanan Wilson

**Affiliations:** Ottawa Hospital Research Institute, Canada

Thank you for your review of our article.

 We agree with the reviewer that *exceedingly *should be removed from the manuscript, and we will do this.

 The definition of a rare disease varies by country (i.e. US <200,000, Europe, <5 in 10,000). The prevalence of hemoglobinopathies are much higher in African then the rest of the world (10.74 vs. 2.73 per 1000) (Fattoum, Mediter J of Hematol and Infect Dis, 2009).

 We have conducted a cost effectiveness analysis (Colyle et al, under review) of our previous study in Bangladesh (Murphy et al, eLife, 2019). This paper is currently under review, and we will add details to the discussion when it is published. There are a number of things to consider when considering different options for preterm birth surveillance including cost and health outcomes. Ultimately the decision about whether to undertake such an initiative will be up to policy makers and global funders. Implementation studies such as this will inform governments and global health funders into the usefulness of these initiatives.

 G6PDH is not on the disease panel in Ontario, which is why it was not included in this study.
